# Evaluation of standard breast ultrasonography by adding two-dimensional and three-dimensional shear wave elastography: a prospective, multicenter trial

**DOI:** 10.1007/s00330-023-10057-9

**Published:** 2023-08-30

**Authors:** Jinshun Xu, Lei Zhang, Wen Wen, Yushuang He, Tianci Wei, Yanling Zheng, Xiaofang Pan, Yuhong Li, Yiyun Wu, Fenglin Dong, Heqing Zhang, Wen Cheng, Hongchun Xu, Yingchun Zhang, Lingyun Bao, Xinguo Zhang, Shichu Tang, Jintang Liao, Honghao Luo, Haina Zhao, Jiawei Tian, Yulan Peng

**Affiliations:** 1 Department of Ultrasound Medicine, Institute of Ultrasound Medicine, West China Hospital of Sichuan University, Chengdu, China; 2 Department of Ultrasound Medicine & Laboratory of Translational Research in Ultrasound Theranostics, Sichuan Clinical Research Center for Cancer, Sichuan Cancer Hospital & Institute, Sichuan Cancer Center, Affiliated Cancer Hospital of University of Electronic Science and Technology of China, Chengdu, China; 3 Department of Ultrasound, the Second Affiliated Hospital of Harbin Medical University, Harbin, China; 4 Department of Medical Ultrasonics, Institute of Diagnostic and Interventional Ultrasound, The First Affiliated Hospital of Sun Yat-Sen University, Guangzhou, China; 5 Health Medical Department, Dalian Municipal Central Hospital, Dalian, China; 6 Department of Ultrasound, The First Affiliated Hospital of Jinzhou Medical University, Jinzhou, China; 7 Department of Ultrasound, Jiangsu Province Hospital of Chinese Medicine, Nanjing, China; 8 Department of Ultrasound, The First Affiliated Hospital of Soochow University, Suzhou, China; 9 Department of Ultrasound, Harbin Medical University Cancer Hospital, Harbin, China; 10 Department of Ultrasound, Shengjing-Dalian Hospital, Chinese Medical Sciences University, Dalian, China; 11 Department of Ultrasound, The Second Affiliated Hospital of Soochow University, Suzhou, China; 12 Department of Ultrasound, Affiliated Hangzhou First People’s Hospital, Zhejiang University School of Medicine, Hangzhou, China; 13 Department of Ultrasound, Shaoyang Central Hospital, Shaoyang, China; 14 Department of Ultrasound, Hunan Provincial Tumor Hospital, Changsha, China; 15 Department of Ultrasound, Xiangya Hospital of Central South University, Changsha, China

**Keywords:** Ultrasonography, Elasticity imaging techniques, Biopsy, Breast neoplasms

## Abstract

**Objective:**

To reduce the number of biopsies performed on benign breast lesions categorized as BI-RADS 4–5, we investigated the diagnostic performance of combined two-dimensional and three-dimensional shear wave elastography (2D + 3D SWE) with standard breast ultrasonography (US) for the BI-RADS assessment of breast lesions.

**Methods:**

A total of 897 breast lesions, categorized as BI-RADS 3–5, were subjected to standard breast US and supplemented by 2D SWE only and 2D + 3D SWE analysis. Based on the malignancy rate of less than 2% for BI-RADS 3, lesions assessed by standard breast US were reclassified with SWE assessment.

**Results:**

After standard breast US evaluation, 268 (46.1%) participants underwent benign biopsies in BI-RADS 4–5 lesions. By using separated cutoffs for upstaging BI-RADS 3 at 120 kPa and downstaging BI-RADS 4a at 90 kPa in 2D + 3D SWE reclassification, 123 (21.2%) participants underwent benign biopsy, resulting in a 54.1% reduction (123 versus 268).

**Conclusion:**

Combining 2D + 3D SWE with standard breast US for reclassification of BI-RADS lesions may achieve a reduction in benign biopsies in BI-RADS 4–5 lesions without sacrificing sensitivity unacceptably.

**Clinical relevance statement:**

Combining 2D + 3D SWE with US effectively reduces benign biopsies in breast lesions with categories 4–5, potentially improving diagnostic accuracy of BI-RADS assessment for patients with breast lesions.

**Trial registration:**

ChiCTR1900026556

**Key Points:**

*• Reduce benign biopsy is necessary in breast lesions with BI-RADS 4–5 category.*

*• A reduction of 54.1% on benign biopsies in BI-RADS 4–5 lesions was achieved using 2D + 3D SWE reclassification.*

*• Adding 2D + 3D SWE to standard breast US improved the diagnostic performance of BI-RADS assessment on breast lesions: specificity increased from 54 to 79%, and PPV increased from 54 to 71%, with slight loss in sensitivity (97.2% versus 98.7%) and NPV (98.1% versus 98.7%).*

**Supplementary information:**

The online version contains supplementary material available at 10.1007/s00330-023-10057-9.

## Introduction

As one of the most common imaging modalities for evaluating breast lesions in women, ultrasonography (US) combined with the Breast Imaging Reporting and Data System (BI-RADS) provides standardized terminology and criteria to assess breast cancer risk [1, 2], based on the description emphases of margin, shape, orientation, and internal and posterior characteristics, together with and without distortion and edema of surrounding tissues [3]. Following this assessment system offers many benefits, including high sensitivity (over 90%) and high reliability and reproducibility (over 90%) [4, 5]. However, diagnostic biopsy is necessary for BI-RADS 4–5 due to the potential risk of malignancy [6]. Unfortunately, biopsies with eventually benign pathology are sometimes inevitable on parts of these lesions, despite the relatively low likelihood of malignancy (ranging from 2 to over 95% depending on the specific category). To reduce the number of benign biopsies in BI-RADS category, optimizing the BI-RADS characterization on US is an important consideration for breast diagnosis.

Shear wave elastography (SWE) is a technique that measures the elasticity of breast tissues, which has been developed to improve the BI-RADS evaluation of breast lesions on US [7–11]. Recent studies have shown that combining B-mode US with SWE improves the specificity of breast US without compromising sensitivity, and it has led to a reduction in benign biopsies for BI-RADS 4–5 breast lesions [12–16]. However, limitations in SWE measurements have been noted due to the high heterogeneity of breast lesions and the two-dimensional (2D) assessment approach. To address these limitations, a three-dimensional (3D) SWE technique has been developed, which allows for automatic mechanical sweep of the ultrasound beam and fully automated 3D elastographic reconstruction of breast lesion stiffness. Combining 2D and 3D SWE (2D + 3D SWE) provides additional elasticity measurements on the coronal plane of breast lesions, leading to a more accurate evaluation [17–19]. This prospective multicenter study aims to investigate the diagnostic performance of the 2D + 3D SWE approach in combination with standard breast US for BI-RADS assessment of breast lesions in women. The hypothesis is that the use of 2D + 3D SWE will improve the accuracy of BI-RADS assessment of breast lesions on US. The results of this study could have important implications for clinical practice and may lead to improved diagnostic accuracy and biopsy efficiency.

## Methods

This prospective multicenter study was conducted between September 2019 and August 2020 and approved by the institutional review board of each recruiting site. Written informed consent was obtained from all participants. Fourteen public general hospitals across 8 provinces in China, where standard breast US, 2D SWE, and 3D SWE were routinely used for breast lesion evaluations, participated in this study. All US examinations were performed using the Aixplorer US system (SuperSonic Imagine), equipped with a linear transducer (SL15-4) and a volume transducer (SLV 16-5). Investigators at each recruiting site received instructions regarding the study protocol, including eligibility criteria, standardized data acquisition, and interpretation procedures before the start of enrollment [20].

### Participants

Inclusion criteria were as follows: (a) adult women who were 18 years of age or older with breast lesions; (b) lesions were recommended for regular observation during short interval follow-up or biopsy; and (c) the largest diameters of lesions were ≥ 5 and ≤ 40 mm. Exclusion criteria included the following: (a) lesions with ipsilateral breast resection or biopsy; (b) lesions assessed as BI-RADS category 0, 1, 2, or 6 on US assessment; (c) lesions located behind the nipple, distance of ≤ 5 or ≥ 40 mm to the skin surface; (d) women who were treated by chemotherapy, radiation, targeted therapy, or endocrine therapy; and (e) women with breast implants or who were pregnant or lactating.

To avoid bias in the recruitment, only an index lesion was enrolled for each participant. The index lesion was defined as the lesion with the highest BI-RADS category on standard breast US. If two or more lesion with equally high BI-RADS category, the largest one was designated the index lesion.

### Standard breast US

Radiologists who had 2–18 years of experience with grayscale US and color Doppler flow imaging and 1–3 years of experience with SWE for breast diagnoses performed the US examination at each site. Sonographic features of lesions were documented in two orthogonal planes (transverse and longitudinal sections) according to the BI-RADS category system [21]. The final results of categories 3 to 5 were eligible to participate in the study.

Grayscale US and color Doppler US were performed by using standardized parameter settings, including repetition frequency between 7 and 10 MHz, general gain of 40–60 dB, depth of region of interest (ROI) between 3 and 4 cm, focus on 2–3 cm tissue zone in depth, dynamic range of less than 60 dB, and color Doppler scale of 6 cm/s. After standard breast US examination, two orthogonal grayscale images in transverse and longitudinal sections and a color Doppler image showing rich blood flow were acquired, and tumor sizes in length, depth, and width were documented.

### 2D and 3D SWE imaging

2D SWE images showed the largest diameter in two orthogonal planes and 3D SWE images performed along the transverse section were acquired at least twice. The representative data with higher image quality were determined by a radiologist [22]. Standardized parameters of 2D SWE and 3D imaging were predetermined as described in the previous studies [19, 23]. Briefly, the transducer was laid on skin without any additional pressure for at least 10 s. Default maximum setting of 180 kilopascals (kPa) on elastic modulus was fixed at each pixel. Quantitative measurements were performed by three times using a 2 mm^2^ region of interest (Q-box). For 3D SWE, three orthogonal planes (axial, sagittal, and coronal) were reconstructed on a 3D image. To quantitatively evaluate lesion elasticity, the stiffest part in the coronal plane of the 3D image was focused and segmented into 4 × 4 slices automatically based on image processing software in the system. Mean elasticities (*E*_mean_) of the Q-box in 2D SWE and 3D SWE were recorded for analysis. An example of 3D reconstruction and segmentation is presented in Supplementary Figures S1 and S2.

For quality control, four experienced SWE specialists (SY.H., JS.X., L.Z., and TC.W.) reviewed all SWE images to exclude images with poor quality and data with inadequate measurement, based on previously described assessment [22, 24]. During follow-up, a data monitoring board was established to monitor the study progress every 6 months.

### Study design

**Fig. 1:**
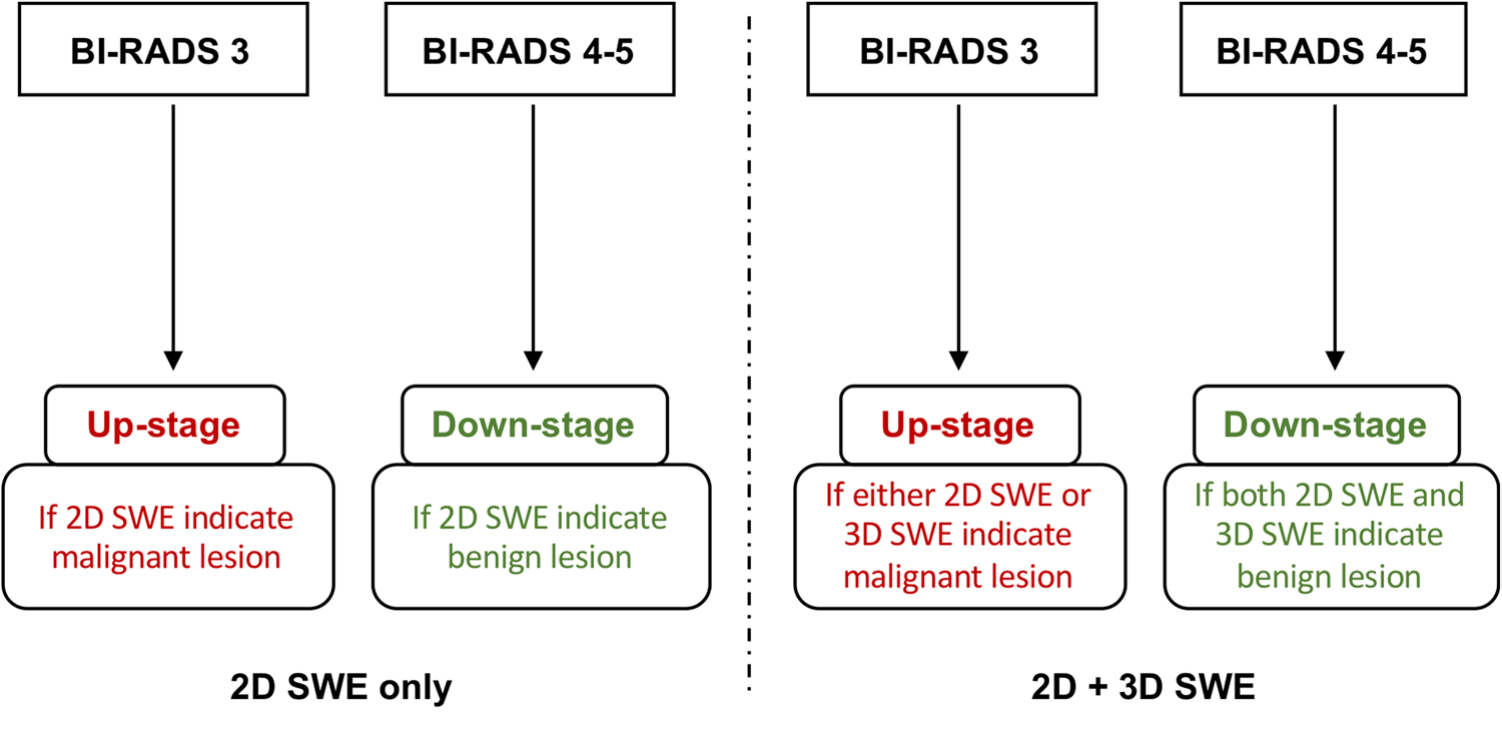
Study design for the reclassification of breast lesions with two-dimensional (2D) and three-dimensional (3D) shear wave elastography (SWE)

For reevaluation of BI-RADS category of breast lesions with an initial category of 3 to 5, the combination of 2D + 3D SWE with standard breast US was used to reevaluate the BI-RADS category of breast lesions with an initial category of 3 to 5. The study design was as follows (Fig. 1): if a lesion with initial BI-RADS 3 category was indicated as benign by both 2D SWE and 3D SWE evaluations, no change remained on the classification; if the lesion was assessed as malignant by either 2D SWE or 3D SWE, it was upstaged into category 4a. Conversely, if a lesion with initial BI-RADS 4–5 category was indicated a benign lesion by both 2D SWE and 3D SWE evaluations, it was downgraded by one step (i.e., 5 to 4c, 4c to 4b, 4b to 4a, and 4a to 3), and no change remained if either 2D SWE or 3D SWE indicated a malignant lesion. The reevaluation was also performed using 2D SWE only for comparative analysis.

### Outcomes and reference standards

Primary outcome was the incidence of benign biopsy, which was defined as biopsies in benign breast lesions with initial BI-RADS 4–5 category using the equation bellow:

$$\mathrm{Benign}\ \mathrm{Biospy}\ \mathrm{Rate}={~}^{{N}_1}\!\left/ \!{~}_{{N}_0}\right.\times 100\%$$

where *N*_1_ presented the total number of benign lesions in BI-RADS 4–5 category, and *N*_0_ presented the total number of benign lesions in the whole study, in order to evaluate the potential reduction of benign biopsies in BI-RADS 4–5 lesions as assessed by standard breast US after reclassifying with 2D + 3D SWE or 2D SWE only. Ultrasound-guided percutaneous puncture with an 18-gauge Bard needle was done to obtain tissue samples for histopathologic evaluation.

Reference standard for the final diagnosis involved a biopsy of malignant lesions and 2-year follow-up of benign lesions. For lesions with BI-RADS 4–5 category by standard breast US examination, histopathologic evaluation was performed. For BI-RADS 3 lesions upgraded to BI-RADS 4a after reclassification with a cutoff of 160 kPa on 2D SWE as previously reported [14], biopsy was performed. For BI-RADS 3 lesions with an increase in size during the 2-year follow-up, biopsy was performed. The probably benign BI-RADS 3 lesions with resolved, decreased, or stable in size during the 2-year follow-up were considered negative.

### Statistical analysis

To evaluate the diagnostic performance of adding 2D SWE and 3D SWE, sorts of cutoffs were used as the criteria for differential identification (benign or malignant) of breast lesions for upstage BI-RADS 3 and downstage BI-RADS 4–5 lesions. Firstly, an independent cutoff that resulted in the same number of malignancies in BI-RADS 3 as standard breast US was used for reclassification with 2D SWE only and 2D + 3D SWE. Secondly, an independent cutoff that resulted in a maximum of 2% malignancies (analogous to BI-RADS 3 definition) was used for SWE reclassifications. Thirdly, separated cutoffs were used for SWE reclassifications to upstage BI-RADS 3 and downstage BI-RADS 4a based on previous suggestions by Berg [14] and Lee [13]. Lastly, separated cutoffs resulting in a maximum of 2% malignancy were used for SWE reclassifications. Malignancy rates in BI-RADS 3 and benign biopsies in BI-RADS 4–5 lesions were measured in each approach and analyzed to assess the diagnostic performance of standard breast US with SWE reclassifications.

## Results

### Participant demographics and characterization

**Fig. 2:**
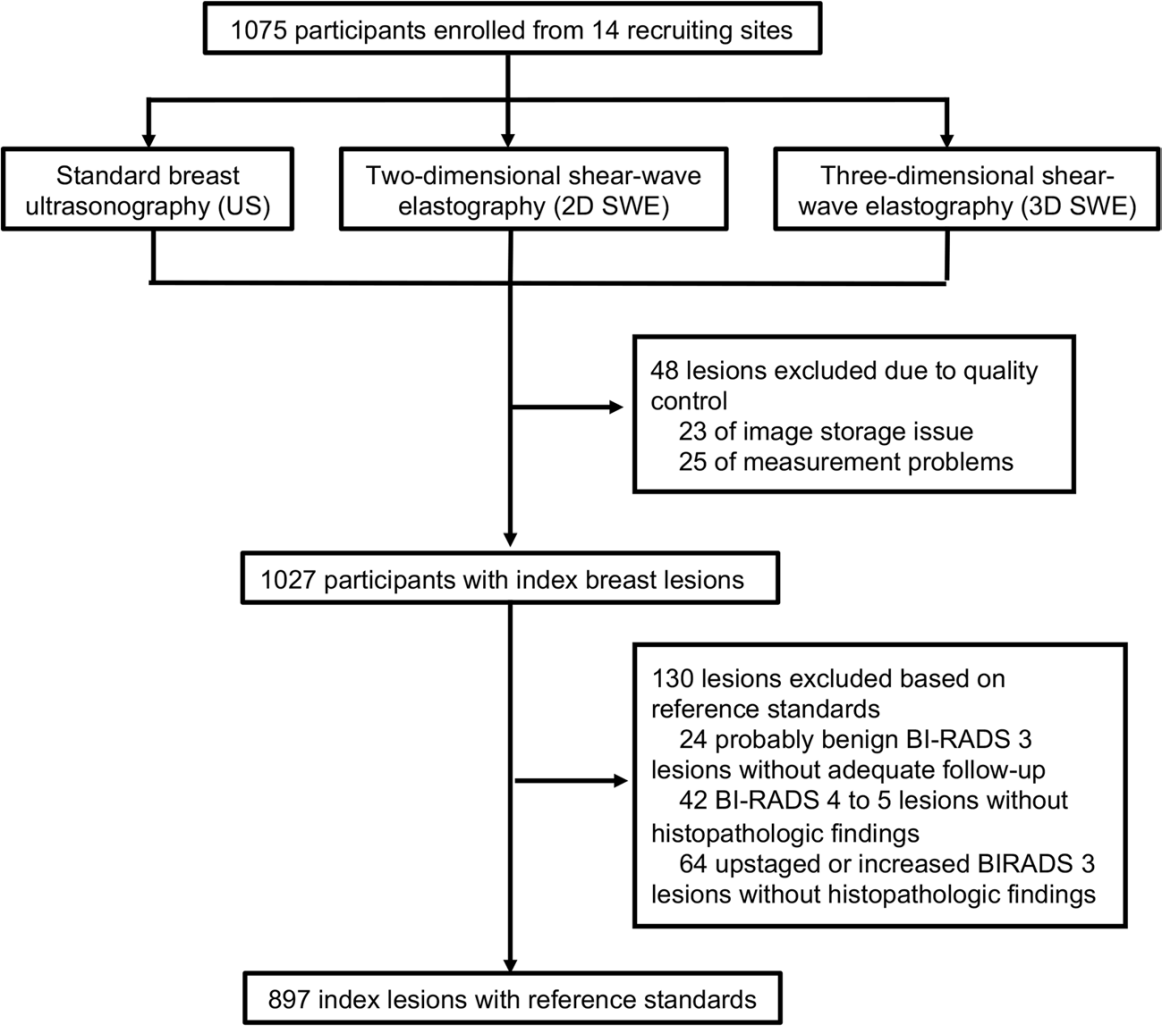
Flow diagram of participant enrollment in the study

A total of 1075 participants were eligible to accept standard breast US, 2D SWE, and 3D SWE examinations (Fig. 2). Forty-eight (4%) participants were dropped out from the data analysis as a result of image storage issues (*n* = 23) and measurement problems (*n* = 25) during the quality control procedure. A hundred and thirty (12%) participants were excluded from reference standards because of inadequate follow-up in probably benign lesions (*n* = 24) and no histopathologic findings in BI-RADS 4 to 5 lesions (*n* = 42), in upstaged BI-RADS 3 lesions after reclassification (*n* = 33), and in size-increased BI-RADS 3 lesions during follow-up (*n* = 31). Eventually, 897 index breast lesions with 581 (64.8%) benign and 316 (35.2%) malignant lesions were enrolled in this multicenter prospective study. The median age of participants was 46 years, with an interquartile range (IQR) between 37 and 54 years. Baseline of demographics and characteristics in the study is summarized in Table S1, findings in each study site are listed in Table S2, and measurement distributions of SWE for benign and malignant lesions are shown in Table S3.

### Diagnostic performance of combined SWE reclassifications

Based on the assessment with standard breast US of 897 lesions, 317 (35.3%) were categorized as BI-RADS 3, 259 (28.9%) as BI-RADS 4a, 104 (11.6%) as BI-RADS 4b, 129 (14.4%) as BI-RADS 4c, and 88 (9.8%) as BI-RADS 5. 59.3% (188 of 317) of lesions in the BI-RADS 3 category were biopsied including 45 lesions upgraded to BI-RADS 4a after reclassification and 143 lesions with an increase in size during the 2-year follow-up, resulting in 97.9% (184/188) of benignity and 2.1% (4/188) of malignancy in biopsy. The incidence of malignancy in BI-RADS 3, 4a, 4b, 4c, and 5 was 1.3% (4 of 317), 14.7% (38 of 259), 73.1% (76 of 104), 89.1% (115 of 129), and 94.3% (83 of 88), respectively. As a result, 1.3% (4 of 317) of malignancy in BI-RADS 3 lesions and 46.1% (268 of 581) of benign biopsies in BI-RADS 4 to 5 lesions were found. The diagnostic performance of standard breast US with and without combination of 2D + 3D SWE and 2D SWE only is shown in Table 1.

**Table 1:** Comparison of diagnostic performance between standard breast ultrasonography assessment and after reclassification with 2D + 3D SWE or 2D SWE only

	Malignancies in BI-RADS 3	Benign biopsies in all	Reduction of benign biopsies compared to standard breast US	Sensitivity	Specificity	PPV	NPV
Standard breast ultrasonography (US)	1.3% (4/317)	46.1% (268/581)		98.7% (312/316)	53.9% (313/581)	53.8% (312/580)	98.7% (313/317)
2D SWE—preserved number of malignancies in BI-RADS 3							
Downstage BI-RADS 4a to 3 with 31.4 kPa	1.1% (4/373)	**36.4% (212/581)**	**20.9% (212 versus 268)**	98.7% (312/316)	63.5% (369/581)	59.5% (312/524)	98.9% (369/373)
Downstage BI-RADS 4a + 4b by one step with 19.1 kPa	1.2% (4/335)	43.0% (250/581)					
Downstage BI-RADS 4a to 3 and upstage BI-RADS 3 to 4a with 35.0 kPa	1.9% (4/205)	65.4% (380/581)					
Downstage BI-RADS 4a + 4b by one step and upstage BI-RADS 3 to 4a with 26.0 kPa	3.3% (4/120)	80.0% (465/581)					
Downstage BI-RADS 4a + 4b + 4c by one step and upstage BI-RADS 3 to 4a with 23.0 kPa	4.9% (4/82)	86.6% (503/581)					
Downstage BI-RADS 4 + 5 by one step and upstage BI-RADS 3 to 4a with 23.0 kPa	4.9% (4/82)	86.6% (503/581)					
2D SWE—malignancy rate of less than 2% in BI-RADS 3							
Downstage BI-RADS 4a to 3 with 37.0 kPa	2.0% (8/400)	**32.5% (189/581)**	**29.5% (189 versus 268)**	97.5% (308/316)	67.5% (392/581)	62.0% (308/497)	98.0% (392/400)
Downstage BI-RADS 4a + 4b by one step with 24.0 kPa	2.0% (7/356)	39.9% (232/581)					
Downstage BI-RADS 4a to 3 and upstage BI-RADS 3 to 4a with 35.0 kPa	1.9% (4/205)	65.4% (380/581)					
2D + 3D SWE—malignancy rate of less than 2% in BI-RADS 3							
Downstage BI-RADS 4a to 3 with 76.0 kPa	2.0% (9/458)	**22.7% (132/581)**	**50.7% (132 versus 268)**	97.2% (307/316)	77.3% (449/581)	69.9% (307/439)	98.0% (449/458)
Downstage BI-RADS 4a + 4b by one step with 42.0 kPa	1.9% (7/369)	37.7% (219/581)					
2D SWE—reclassification with separated cutoffs							
Upstage BI-RADS 3 with a cutoff of 160 kPa, downstage BI-RADS 4a with a cutoff of 30 kPa	0.8% (3/367)	37.3% (217/581)					
Upstage BI-RADS 3 with a cutoff of 160 kPa, downstage BI-RADS 4a with a cutoff of 80 kPa	3.5% (18/513)	14.8% (86/581)					
Upstage BI-RADS 3 with a cutoff of 120 kPa, downstage BI-RADS 4a with a cutoff of 50 kPa	1.9% (8/421)	28.9% (168/581)	37.3% (168 versus 268)				
2D + 3D SWE—reclassification with separated cutoffs							
Upstage BI-RADS 3 with a cutoff of 160 kPa, downstage BI-RADS 4a with a cutoff of 30 kPa	1.0% (3/302)	48.5% (282/581)					
Upstage BI-RADS 3 with a cutoff of 160 kPa, downstage BI-RADS 4a with a cutoff of 80 kPa	1.9% (8/430)	27.4% (159/581)					
Upstage BI-RADS 3 with a cutoff of 120 kPa, downstage BI-RADS 4a with a cutoff of 90 kPa	1.9% (9/467)	**21.2% (123/581)**	**54.1% (123 versus 268)**	97.2% (307/316)	78.8% (458/581)	71.4% (307/430)	98.1% (458/467)

Bold values present the best meaningful results (benign biopsy rate and benign biopsy reduction) in different methods, such as using 2D SWE and 2D+3D SWE with dependent cutoff and separated cutoffs based on the preserved number of malignancies in BI-RADS 3, or keeping malignancy rate of less than 2% in BI-RADS 3, respectively

When 2D SWE only was added to the standard breast US examination at a cutoff of 31.4 kPa, reclassification of BI-RADS 4a lesions to BI-RADS 3 resulted in the same number of malignancies (4) in BI-RADS 3 as the standard breast US, and the number of benign biopsies in BI-RADS 4–5 lesions was reduced by 20.9% (212 versus 268). Keeping the malignancy rate of less than 2% in BI-RADS 3, reclassification of BI-RADS 4a to BI-RADS 3 by adding 2D SWE only with a cutoff of 37 kPa resulted in a reduction of 29.5% (189 versus 268) in benign biopsies.

Compared to reclassification of BI-RADS 4a to BI-RADS 3, reclassifying BI-RADS 4a+4b, BI-RADS 3+4a, BI-RADS 3+4a+4b, BI-RADS 3+4a+4b+4c, and BI-RADS 3+4+5 lesions resulted in an increase in the number of benign biopsies, when using the two approaches of either preserving the malignancy number or preserving malignancy rate in BI-RADS 3 on adding 2D SWE only or 2D + 3D SWE (different cutoffs used are shown in Table 1).

Compared to 2D SWE only reclassification, 2D + 3D SWE reclassification of BI-RADS 4a to BI-RADS 3 at a cutoff of 76.0 kPa resulted in 2.0% (9 of 458) missed cancers and 22.7% (132 of 581) benign biopsies. Reduction of benign biopsies in BI-RADS 4–5 lesions reached to 50.7% (132 versus 268). When using separated cutoffs by upstage BI-RADS 3 at a cutoff of 120 kPa and downstage BI-RADS 4a at a cutoff of 90 kPa, an optimum 54.1% (123 versus 268) reduction with 21.2% (123 of 581) benign biopsies and 1.9% (9 of 467) missed cancers was achieved. The malignancy rate in BI-RADS 4a lesions was increased from 14.7% (38/259) to 30.3% (33/109) after using the separated cutoffs.

### 2D + 3D SWE analysis of histopathologic subtypes

**Fig. 3:**
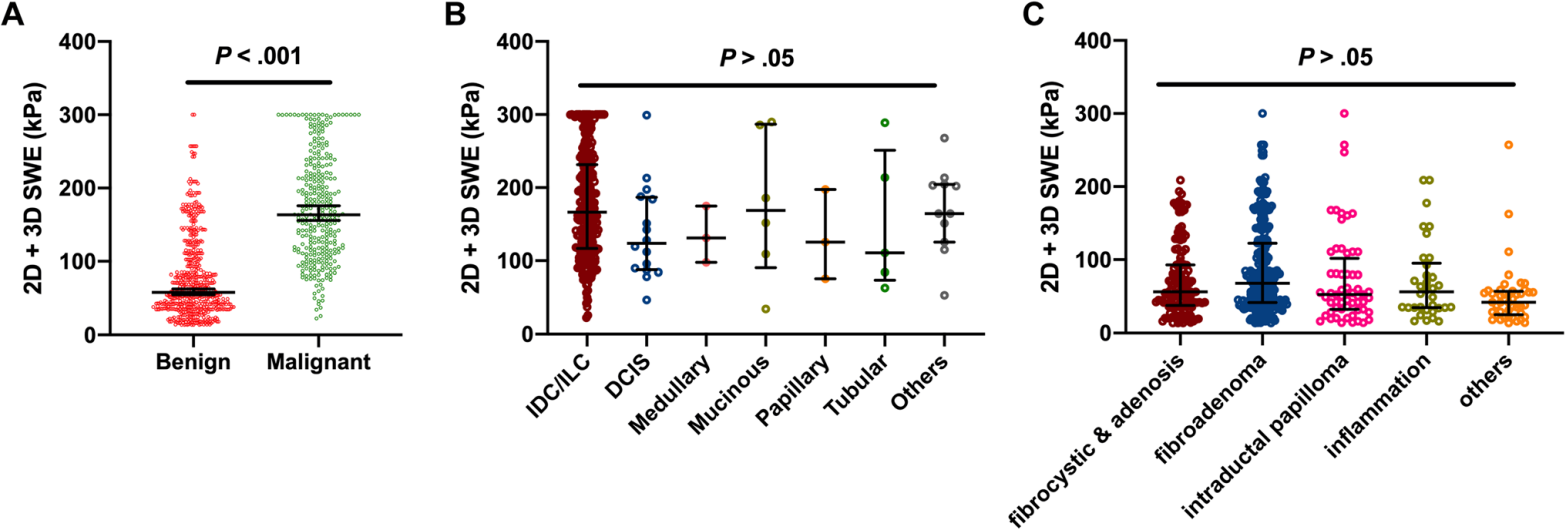
Shear wave elastography (SWE) value analyses of benign and malignant lesions by two-dimensional SWE and three-dimensional SWE (2D + 3D SWE) assessment. Data are expressed as the median (25th, 75th percentile). *p* < .001 between benign and malignant lesions (**A**) by Welch’s two independent sample *t*-test. *p* > .05 among histopathologic subtypes of malignant (**B**) and benign (**C**) lesions by one-way ANOVA using Tukey’s multiple comparisons test. IDC invasive ductal carcinoma, ILC invasive lobular carcinoma, DCIS ductal carcinoma in situ

Malignant lesions exhibited higher values in *E*_mean_ than benign lesions (median 163.6 versus 58.2, *p* < .001) (Fig. 3A). No significant differences were found among the subtypes of malignant and benign lesions, respectively (Fig. 3B, C).

After 2D + 3D SWE reclassification at separated cutoffs (upstage BI-RADS 3 at 120 kPa and downstage BI-RADS 4a at 90 kPa), nine malignancies were missed in BI-RADS 3 category including three invasive ductal/lobular carcinomas (IDC/ILC), three ductal carcinomas in situ (DCIS), two papillary carcinomas, and one tubular carcinoma. Detailed molecular types of these malignancies are shown in Table 2.

**Table 2:** Undetected malignancies by combined 2D + 3D SWE reclassification with separated cutoffs using upstaging BI-RADS 3 at 120 kPa and downstaging BI-RADS 4a at 90 kPa

Cases	Age (year)	Longest size in diameter (mm)	2D + 3D SWE (kPa)	Pathology	Grade	Molecular typing	HER2	ER (%)	PR (%)	Ki-67 (%)
1	41	31.6	42.2	IDC	3	HER-2 +	++	90	80	5
2	42	11.3	59.8	DCIS	2	HER-2 +	+++	70	90	10
3	50	6.1	68.4	Papillary	1	TNBC	-	0	0	2
4	36	14.8	74.7	ILC	1	HER-2 +	++	30	0	30
5	37	18.5	76.0	Papillary	3	Luminal B	-	20	10	5
6	34	11.6	81.4	IDC	2	Luminal B	-	5	0	80
7	28	10.7	84.6	Tubular	2	Luminal A	-	80	80	20
8	48	5.4	84.6	DCIS	1	HER-2 +	++	0	0	30
9	42	5.0	89.8	DCIS	2	Luminal A	-	85	85	20

*IDC* invasive ductal carcinoma, *ILC* invasive lobular carcinoma, *DCIS* ductal carcinoma in situ, *TNBC* triple negative breast carcinoma, *HER2* human epidermal growth factor receptor type 2, *PR* progesterone receptor, *ER* estrogen receptor

### Measurement reliability of SWE

A reasonably strong agreement (Pearson correlation, 0.86–0.95) in the reliability of SWE measurements was observed in three orthogonal planes of 2D + 3D SWE examinations (Table 3).

**Table 3:** Measurement reliability of SWE values in three orthogonal planes

	Pearson correlations		
	*E*_axial_	*E*_sagittal_	*E*_coronal_
M_1_ versus M_2_	0.89	0.87	0.86
M_1_ versus M_3_	0.88	0.91	0.89
M_2_ versus M_3_	0.95	0.92	0.88

M_1_, M_2_, and M_3_ present three SWE measurements. *E*_axial_ and *E*_sagittal_ indicate the lesion elasticity values measured in the axial and sagittal planes of 2D SWE; *E*_coronal_ indicates the values measured in the coronal plane of 3D SWE

### Representative SWE images

**Fig. 4:**
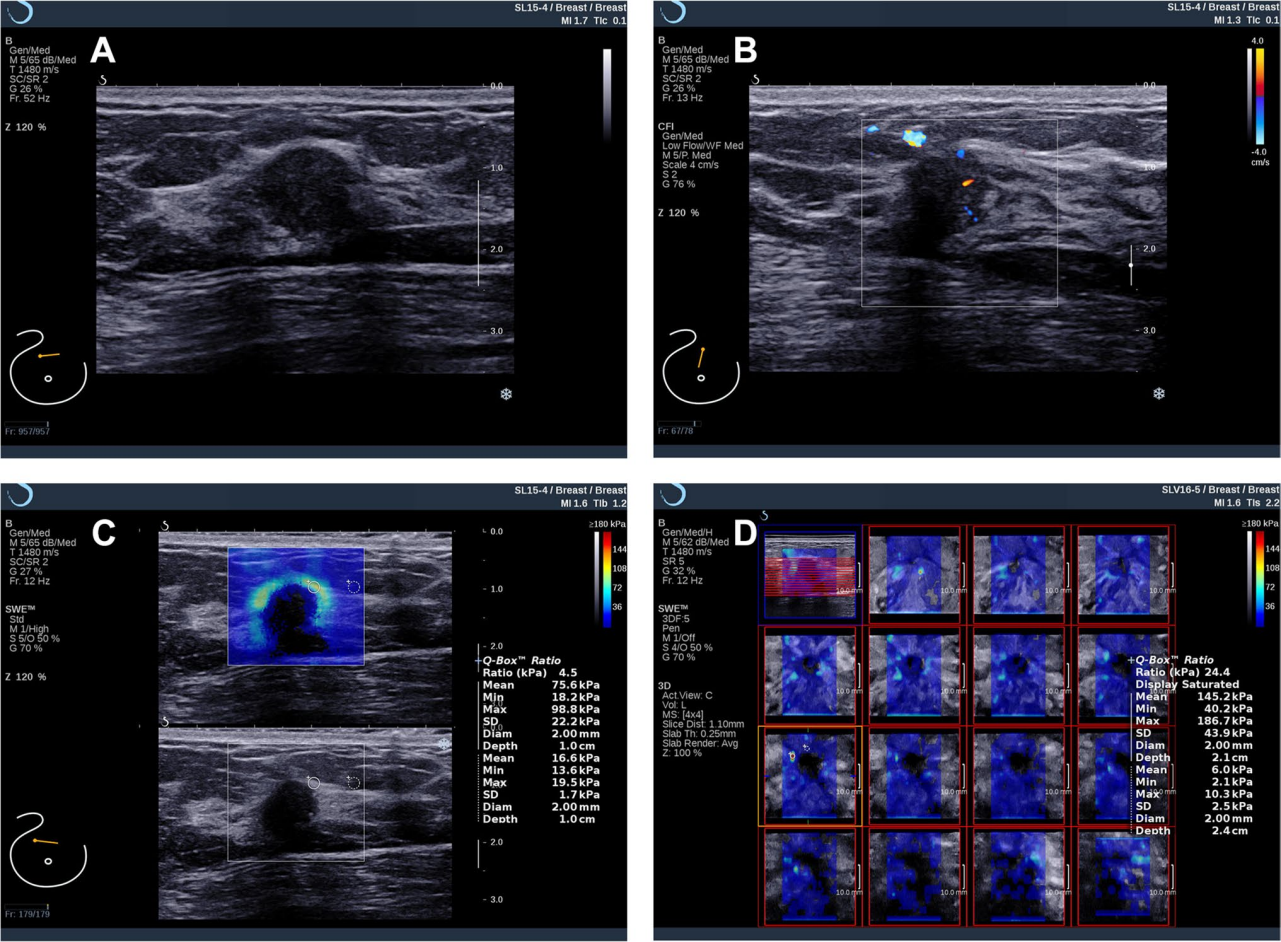
A 55-year-old female participant with papillary cancer in the right breast. BI-RADS 3 was assessed by standard breast US examination using a B-mode ultrasound image (**A**) and a color Doppler image (**B**). **C** A mean elasticity of 75.6 kPa in the 2D SWE only assessment indicated no change in the BI-RADS category. **D** A mean elasticity of 145.2 kPa in 2D + 3D SWE assessment indicated that upstaging to BI-RADS 4a should be carried out

**Fig. 5:**
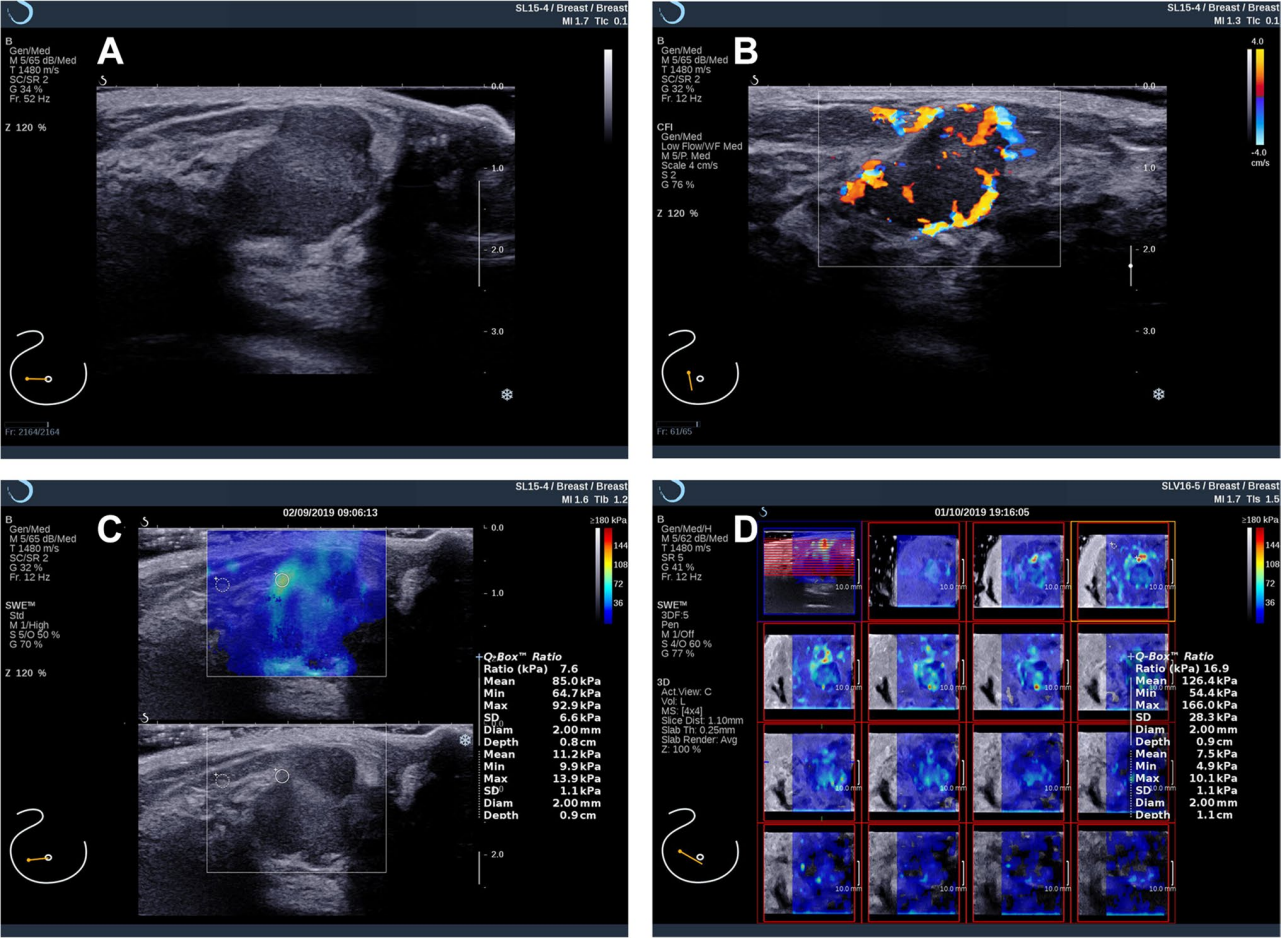
A 46-year-old female participant with ductal carcinomas in situ (DCIS) in the right breast. Standard breast US assessed to BI-RADS 4a using B-mode ultrasound (**A**) and color Doppler (**B**) examination. **C** A mean elasticity of 85.0 kPa according to 2D SWE only indicated downstaging of the lesion to BI-RADS 3. **D** A mean elasticity of 126.4 kPa in 2D + 3D SWE assessment indicated that biopsy should be performed in this lesion

A lesion with BI-RADS 3 category by standard breast US showed false-negative in 2D SWE only (*E*_mean_ < 120 kPa) and true-positive in 2D + 3D SWE (*E*_mean_ > 120 kPa) (Fig. 4). As a result, this lesion turned out to be papillary cancer (HER2 positive, hormone receptor negative). A DCIS (HER2 positive, hormone receptor positive) with BI-RADS 4a category by standard breast US showed false-negative 2D SWE (*E*_mean_ < 90 kPa) and true-positive 2D + 3D SWE (*E*_mean_ > 90 kPa) (Fig. 5).

## Discussion

This prospective study enrolled 897 index breast lesions with reference standards from 14 hospitals to evaluate whether adding 2D + 3D SWE on standard breast US could improve diagnostic performance of BI-RADS assessment for breast lesions. As a result, 46% (268 of 581) of lesions following standard breast US assessment underwent benign biopsies, and 82% (221 of 268) of benign biopsies occurred in the BI-RADS 4a category, accompanied by low specificity (54%) and low positive predictive value (PPV, 54%) in all. After the addition of 2D SWE only for reclassification, reduction of benign biopsies in BI-RADS 4–5 lesions was 20~30% using an independent cutoff. Simultaneously, specificity and PPV were increased from 54% to approximately 65% and 60%, respectively. These findings demonstrated that the addition of SWE on standard breast US can be used to refine BI-RADS assessment of breast lesions. When 2D + 3D SWE reclassification with an independent cutoff was performed, 50% of reduction on benign biopsies in BI-RADS 4–5 lesions was achieved, and specificity and PPV were increased to 77% and 70%, respectively.

Compared to an independent cutoff, using separated cutoffs (upstage of BI-RADS 3 and downstage of BI-RADS 4a) achieved further improvement on BI-RADS evaluation in both 2D SWE only and 2D + 3D SWE reclassifications. The reduction of benign biopsies in BI-RADS 4–5 lesions on 2D SWE reclassification with separated cutoffs was 37%, 1.2-fold higher than that with an independent cutoff. Ideally, a reduction of 54% on 2D + 3D SWE reclassification using separated cutoffs was obtained with improved specificity (79%) and PPV (71%). All SWE reclassifications with either independent or separated cutoffs mentioned above exhibited considerable stability in sensitivity (97%) and negative predictive value (NPV, 98%).

The underlying rationale originated from an artifact that some very stiff cancers appeared “soft” in SWE images because of the poor signal-to-noise ratio caused by increased noise and decreased shear wave velocity in stiff cancers [7]. 2D + 3D SWE was able to reduce this artifact by acquiring comprehensive elasticity information of lesions from three orthogonal planes. Based on that, the appropriate cutoffs were measured for BI-RADS reclassification to reduce benign biopsies. Since 3D SWE in the system is constructed from 2D SWE, it would be helpful for the comparison of the shear wave speed cutoffs from the various studies in the future.

SWE cutoffs to differentiate benign from malignant breast lesions are still under academic assessment. Specifically, a previous study showed that a SWE cutoff of 2.55 m/s (elasticity value in velocity) was promising on reclassifying BI-RADS 4a lesions [25]. If SWE and strain elastography (SE) were added to routine B-mode breast ultrasound, an SWE cutoff at 3.77 m/s was suggested [16]. Considering much evidence on elastography as an adjuvant for breast diagnosis, use of an independent cutoff is easily carried out but might not accurately reflect the prospect of individual diagnosis. Two cutoffs or more were worthy of consideration. Berg et al indicated that use of a cutoff of 80 kPa for downgraded BI-RADS 4a and a cutoff of 160 kPa for upgraded BI-RADS 3 was effective in improving specificity of breast US assessment without loss of sensitivity [14]. Afterwards, cutoffs of 30 kPa for downgraded BI-RADS 4a and 160 kPa for upgraded BI-RADS 3 were reported to increase specificity of screening US of dense breasts [13]. However, to the best of our knowledge, no separated cutoffs have been used to reduce the benign biopsies. This study first explored the feasibility of using separated cutoffs for up/downgrade suspicious breast lesions on 2D + 3D SWE reclassification. As a result, a 54% reduction of benign biopsies in BI-RADS 4–5 lesions was obtained at separated cutoffs of 120 kPa for upgrading BI-RADS 3 and 90 kPa for downgrading BI-RADS 4a.

In comparison with reclassifying BI-RADS 4a lesions with an independent cutoff and reclassifying BI-RADS 3 and 4a lesions with separated cutoffs, reclassification of BI-RADS 4b, 4c, and 5 with SWE provided no benefit on reduction of benign biopsies, suggesting that women with breast lesions in BI-RADS 4b, 4c, and 5 were unlikely to benefit from this approach. Similar results were also discussed by Golatta [16].

Our study illustrated that higher SWE values of lesion resulted in higher possibility of malignancy, consistent with previous reports [12, 13, 15]. Nevertheless, a few low SWE cancers with low SWE values will be missed. Our study detected 36 cancers with a value of less than 90 kPa in 2D + 3D SWE, and 25% (9/36) of them were missed after reclassification. The pathologic types of missed cancers did not fit very well with previous reports that DCIS and papillary cancers had relatively low values on elastography [15]. Future research may explore the potential relationships between subtypes of breast cancers and elastography characterizations.

Several limitations existed. First, findings of standard breast US, 2D SWE, and 3D SWE were not blinded to each other. Second, quantitative measurement in SWE without qualitative color features was used for elasticity evaluation. Color features of SWE known as high reproductivity should be further investigated [9, 24]. Third, this prospective study focused on the evaluation of 2D + 3D SWE performance combined with standard breast US by hypothetically reclassifying participants into the respective diagnostic categories (BI-RADS 3–5), but we do not yet know the clinical consequences of additional SWE reclassification. A validation cohort for participant care should be investigated. Fourth, audit parameters of 3D SWE examinations for all breast lesions could not be determined because lesions with BI-RADS 3–5 only were included. Fifth, although this study had been prospectively recruited, the optimal elastography cutoffs were experimentally determined after examination completion and differed from the a priori cutoff for upstage of BI-RADS 3 lesions at the start of initial examination process. These cutoffs remain to be prospective validation. Finally, generalizability of this technique was not warranted due to heterogenous variance of data acquirement and one apparatus only was used, although a panel of four experts had elected for quality control of images and data acquisition.

## Conclusions

Adding combined 2D SWE and 3D SWE to standard breast US for reclassification of breast lesions could reduce benign biopsies in BI-RADS 4–5 lesions by approximately 54% while keeping the malignancy rate in the BI-RADS 3 category less than 2%, using the separated cutoffs at 120 kPa for upstaging of BI-RADS 3 and 90 kPa for downstaging of BI-RADS 4a.

### Supplementary information

ESM 1 (PDF 386 kb)

## Abbreviations

2D SWE: Two-dimensional shear wave elastography
3D SWE: Three-dimensional shear wave elastography
BI-RADS: Breast Imaging Reporting and Data System
DCIS: Ductal carcinoma in situ
ER: Estrogen receptor
HER2: Human epidermal growth factor receptor type 2
ILC: Invasive lobular carcinoma
ITC: Invasive tubular carcinoma
PR: Progesterone receptor

## Declarations

### Guarantor

The scientific guarantor of this publication is Yulan Peng.

### Statistics and biometry

No complex statistical methods were necessary for this paper.

### Study subjects or cohorts overlap

None study subjects or cohorts have been previously reported.

### Methodology

- prospective
- observational
- multicenter study
