# Correction: Evaluation of standard breast ultrasonography by adding two-dimensional and three-dimensional shear wave elastography: a prospective, multicenter trial

**DOI:** 10.1007/s00330-023-10287-x

**Published:** 2023-10-17

**Authors:** Jinshun Xu, Lei Zhang, Wen Wen, Yushuang He, Tianci Wei, Yanling Zheng, Xiaofang Pan, Yuhong Li, Yiyun Wu, Fenglin Dong, Heqing Zhang, Wen Cheng, Hongchun Xu, Yingchun Zhang, Lingyun Bao, Xinguo Zhang, Shichu Tang, Jintang Liao, Honghao Luo, Haina Zhao, Jiawei Tian, Yulan Peng

**Affiliations:** 1 Department of Ultrasound Medicine, Institute of Ultrasound Medicine, West China Hospital of Sichuan University, Chengdu, China; 2 Department of Ultrasound Medicine & Laboratory of Translational Research in Ultrasound Theranostics, Sichuan Clinical Research Center for Cancer, Sichuan Cancer Hospital & Institute, Sichuan Cancer Center, Affiliated Cancer Hospital of University of Electronic Science and Technology of China, Chengdu, China; 3 Department of Ultrasound, the Second Affiliated Hospital of Harbin Medical University, Harbin, China; 4 Department of Medical Ultrasonics, Institute of Diagnostic and Interventional Ultrasound, The First Affiliated Hospital of Sun Yat-Sen University, Guangzhou, China; 5 Health Medical Department, Dalian Municipal Central Hospital, Dalian, China; 6 Department of Ultrasound, The First Affiliated Hospital of Jinzhou Medical University, Jinzhou, China; 7 Department of Ultrasound, Jiangsu Province Hospital of Chinese Medicine, Nanjing, China; 8 Department of Ultrasound, The First Affiliated Hospital of Soochow University, Suzhou, China; 9 Department of Ultrasound, Harbin Medical University Cancer Hospital, Harbin, China; 10 Department of Ultrasound, Shengjing-Dalian Hospital, Chinese Medical Sciences University, Dalian, China; 11 Department of Ultrasound, The Second Affiliated Hospital of Soochow University, Suzhou, China; 12 Department of Ultrasound, Affiliated Hangzhou First People’s Hospital, Zhejiang University School of Medicine, Hangzhou, China; 13 Department of Ultrasound, Shaoyang Central Hospital, Shaoyang, China; 14 Department of Ultrasound, Hunan Provincial Tumor Hospital, Changsha, China; 15 Department of Ultrasound, Xiangya Hospital of Central South University, Changsha, China

**Correction: European Radiology**

**https://doi.org/10.1007/s00330-023-10057-9**

In this article the author name Yiyun Wu was incorrectly written as Yibin Wu.

Furthermore:

- The affiliation details for author Xiaofang Pan were incorrectly given as "Department of Ultrasound, Dalian Central Hospital of Dalian Medical University, Dalian, China" but should have been "Health Medical Department, Dalian Municipal Central Hospital, Dalian, China".

- The affiliation details for author Yiyun Wu were incorrectly given as "Department of Ultrasound, Jiangsu Province Hospital of Traditional Chinese Medicine, Nanjing, China" but should have been "Department of Ultrasound, Jiangsu Province Hospital of Chinese Medicine, Nanjing, China".

- The affiliation details for author Fenglin Dong were incorrectly given as "Department of Ultrasound, The First Affiliated Hospital of Suzhou University, Suzhou, China" but should have been "Department of Ultrasound, The First Affiliated Hospital of Soochow University, Suzhou, China".

- The affiliation details for author Wen Cheng were incorrectly given as "Department of Ultrasound, The Affiliated Cancer Hospital of Harbin Medical University, Harbin, China" but should have been "Department of Ultrasound, Harbin Medical University Cancer Hospital, Harbin, China".

- The affiliation details for author Yingchun Zhang were incorrectly given as "Department of Ultrasound, The Second Affiliated Hospital of Suzhou University, Suzhou, China" but should have been "Department of Ultrasound, The Second Affiliated Hospital of Soochow University,Suzhou, China".

- The affiliation details for author Lingyun Bao were incorrectly given as "Department of Ultrasound, Hangzhou First People’s Hospital of Zhejiang University, Hangzhou, China" but should have been "Department of Ultrasound, Affiliated Hangzhou First People’s Hospital, Zhejiang University School of Medicine, Hangzhou, China".

The original article has been corrected.

