# Supplementary material for: Evaluation of standard breast ultrasonography by adding two-dimensional and three-dimensional shear wave elastography: a prospective, multicenter trial
Source: Eur Radiol. 2023 Aug 30;34(2):945–56. doi: 10.1007/s00330-023-10057-9 (PMC11322273; doi:10.1007/s00330-023-10057-9)

Evaluation of standard breast ultrasonography by adding two-dimensional and three-dimensional shear wave elastography: a prospective, multicenter trial

ELECTRONIC SUPPLEMENTARY MATERIAL

Table S1. Participation baseline on demographics and clinical characteristics

Characteristic	Benign Lesion (n = 581)	Malignant Lesion (n = 316)	Total (n = 897)	*P*-value
**Age (years)**				< .001[t]
median (25th, 75th percentile)	41 (35, 49)	52 (46, 60)	46 (37, 54)	
mean ± SD	42.6 ± 11.4	52.6 ± 10.9	46.1 ± 12.2	
> 40 years				< .001[t]
median (25th, 75th percentile)	49 (45, 57)	54 (48, 61.5)	51 (46, 60)	
mean ± SD	51.5 ± 8.3	55.2 ± 9.0	53.3 ± 8.8	
< 40 years				.014[t]
median (25th, 75th percentile)	35 (29, 38)	37 (33, 39)	35.5 (30, 38)	
mean ± SD	33.6 ± 5.3	35.6 ± 4.7	33.8 ± 5.3	
**Maximum Diameter of lesion (mm)**				< .001[t]
median (25th, 75th percentile)	13 (9, 17.2)	16.5 (12.0, 21.9)	13.9 (10.0, 18.9)	
mean ± SD	13.8 ± 6.2	17.3 ± 6.9	15.0 ± 6.7	
**Histopathologic evaluation No. (%)**				
Benign[*]				
fibrocystic disease and adenosis	93 (16.0)			
fibroadenoma	221 (38.0)			
intraductal papilloma	60 (10.3)			
inflammation	34 (5.8)			
others	44 (7.6)			
N/A	129 (22.2)			
malignant				
IDC/ILC		272 (86.1)		
DCIS		16 (5.1)		
medullary carcinoma		3 (0.9)		
mucinous carcinoma		6 (1.9)		
papillary carcinoma		3 (0.9)		
tubular carcinoma		5 (1.6)		

others	11 (3.5)

IDC = invasive ductal carcinoma, ILC = invasive lobular carcinoma, DCIS = ductal carcinoma in situ

[t] Welch's two independent sample t test.

[*]As for the probably benign BI-RAD 3 lesions without biopsies, the pathological results of these lesions were assorted to N/A (not applicable).

Table S2. Comparison of findings at each study site

	Study 1	Study 2	Study 3	Study 4	Study 5	Study 6	Study 7	Study 8	Study 9	Study 10	Study 11	Study 12	Study 13	Study 14	*P*-value
**Patient No. (%)**	117 (13.0)	236 (26.3)	96 (10.7)	84 (9.4)	30 (3.3)	32 (3.6)	43 (4.8)	32 (3.6)	75 (8.4)	38 (4.1)	26 (2.9)	54 (6.0)	24 (2.7)	10 (1.1)	NA
**Age (years)**															< .05[F]
mean ± SD	48.8 ± 10.8	45.9 ± 11.2	49.5 ± 12.7	39.3 ± 11.1	43.1 ± 10.7	44.1 ± 10.8	53.1 ± 12.0	53.6 ± 9.7	47.4 ± 12.3	44.3 ± 13.1	45.9 ± 16.9	43.6 ± 11.0	36.5 ± 10.0	38.5 ± 10.2	
median (25th, 75th percentile)	47 (42, 56.5)	46 (38, 52)	49 (40, 60)	37 (32, 44)	45 (37, 50)	40 (37, 50)	55 (47, 62)	54 (46, 61)	50 (38, 58)	40 (37, 51)	49 (29, 61)	42 (36, 49)	35 (27, 42)	48 (31, 41)	
**Benign lesions No. (%)**	60 (51.3)	169 (71.6)	45 (46.9)	80 (95.2)	25 (83.3)	29 (90.6)	17 (39.5)	7 (21.9)	50 (66.7)	29 (76.3)	12 (46.2)	23 (42.6)	22 (91.7)	9 (90)	< .05[c,#]
**Malignant lesions No. (%)**	57 (48.7)	67 (28.4)	51 (53.1)	4 (4.8)	5 (16.7)	3 (9.4)	26 (60.5)	25 (78.1)	25 (33.3)	9 (23.7)	14 (53.8)	31 (57.4)	2 (8.3)	1 (10)	
**BI-RADS classification**															
BI-RADS 3 No. (%)	14 (12.0)	56 (23.7)	6 (6.3)	79 (94)	21 (70)	28 (87.5)	0	1 (3.1)	42 (56.0)	26 (70.3)	10 (38.5)	4 (7.4)	21 (87.5)	9 (90)	< .05[c,&]

BI-RADS 4a No. (%)	48 (41.0)	103 (43.6)	35 (36.5)	2 (2.4)	3 (10.0)	1 (3.1)	15 (34.9)	11 (34.4)	15 (20.0)	2 (5.4)	3 (11.5)	19 (35.2)	1 (4.2)	0	
BI-RADS 4b No. (%)	22 (18.8)	14 (5.9)	17 (17.7)	2 (2.4)	1 (3.3)	0	10 (23.3)	10 (31.3)	11 (14.7)	3 (8.1)	2 (7.7)	10 (18.5)	1 (4.2)	1 (10)	
BI-RADS 4c No. (%)	21 (17.9)	38 (16.1)	29 (30.2)	1 (1.2)	2 (6.7)	3 (9.4)	8 (18.6)	2 (6.4)	4 (5.3)	5 (13.5)	8 (30.8)	7 (13.0)	1 (4.2)	0	
BI-RADS 5 No. (%)	12 (10.3)	25 (10.6)	9 (9.4)	0	3 (10.0)	0	10 (23.3)	8 (25.0)	3 (4.0)	1 (2.7)	3 (11.5)	14 (25.9)	0	0	
**2D SWE overall (kPa)**															< .05[F]
mean ± SD	83.1 ± 62.6	95.8 ± 89.0	116.6 ± 91.6	44.6 ± 41.0	86.9 ± 74.7	44.6 ± 31.4	75.5 ± 53.6	110.0 ± 54.4	67.7 ± 53.0	79.9 ± 67.5	113.9 ± 97.5	90.9 ± 69.5	46.3 ± 56.1	57.2 ± 36.1	
median (25th, 75th percentile)	66.3 (39.7, 109.6)	54.8 (34.2, 138.2)	84.3 (37.7, 195.1)	35.1 (21.4, 48.4)	65.4 (33.5, 101.4)	35.7 (21.1, 54.5)	67.9 (33.4, 91.9)	100.1 (75.3, 135.5)	53.7 (30.6, 90.8)	52.9 (32.6, 116.4)	80.5 (24.3, 213.8)	66.0 (38.9, 134.9)	34.8 (21.6, 40.5)	52.2 (26.9, 89.8)	
**2D SWE benign (kPa)**															< .05[F]
mean ± SD	55.2 ± 39.6	54.5 ± 46.6	61.1 ± 59.3	40.2 ± 27.6	63.7 ± 47.8	40.9 ± 30.3	56.4 ± 50.2	60.9 ± 32.9	49.4 ± 30.2	53.6 ± 37.9	28.2 ± 18.9	44.9 ± 33.8	30.5 ± 12.3	57.2 ± 36.1	

median (25th, 75th percentile)	46.8 (23.9, 72.2)	42.2 (23.6, 67.5)	41.3 (30.5, 58.3)	34.9 (21.4, 42.9)	43.2 (29.4, 85.2)	34.7 (19.1, 46.4)	29.2 (20.2, 86.7)	54.9 (33.5, 86.3)	40.3 (24.1, 68.1)	41.0 (29.4, 74.7)	24.2 (15.1, 34.2)	39.1 (23.3, 54.1)	33.9 (20.5, 39.3)	50.8 (25.7, 90.4)	
**2D SWE malignant (kPa)**															< .05[F]
mean ± SD	113.4 ± 69.0	199.8 ± 89.1	165.5 ± 87.4	132.4 ± 125.2	202.4 ± 82.0	80.5 ± 18.0	87.9 ± 53.0	123.8 ± 51.4	108.9 ± 69.1	175.4 ± 66.3	187.3 ± 73.6	124.9 ± 69.8	220.4 ± 55.6	54.2 ± NA	
median (25th, 75th percentile)	97.8 (64.9, 135.9)	204.5 (116.1, 289.9)	159.6 (88.0, 249.0)	112.9 (24.2, 260.1)	191.9 (129.1, 280.9)	74.7 (66.0, NA)	77.7 (55.1, 103.9)	109.5 (81.3, 166.0)	95.1 (55.6, 147.3)	165.4 (115.8, 231.1)	194.1 (118.5, 247.5)	117.3 (65.7, 181.5)	220.4 (181.1, NA)	54.2 (54.2, 54.2)	
**3D SWE overall (kPa)**															< .05[F]
mean ± SD	130.0 ± 48.9	105.2 ± 71.2	116.5 ± 70.6	66.7 ± 58.0	110.9 ± 74.2	48.7 ± 34.1	94.4 ± 45.5	110.6 ± 44.8	67.2 ± 41.6	86.7 ± 67.6	99.7 ± 68.2	114.5 ± 72.1	80.9 ± 64.2	81.1 ± 62.9	
median (25th, 75th percentile)	126.1 (83.5, 163.4)	80.9 (44.7, 167.7)	105.6 (50.9, 179.3)	42.2 (26.5, 77.6)	97.7 (38.0, 173.5)	39.5 (24.2, 60.5)	96.6 (60.2, 115.6)	110.2 (91.6, 138.0)	61.9 (36.2, 90.1)	66.8 (29.7, 144.5)	90.6 (32.2, 155.4)	108.8 (51.2, 172.9)	57.9 (33.2, 127.8)	65.6 (32.1, 126.4)	
**3D SWE benign (kPa)**															< .05[F]
mean ± SD	114.7 ± 51.1	75.3 ± 54.1	68.4 ± 47.5	62.8 ± 55.7	97.3 ± 72.4	42.6 ± 29.4	67.6 ± 44.6	62.4 ± 39.9	57.6 ± 36.1	64.3 ± 57.8	38.1 ± 24.1	62.0 ± 47.4	75.2 ± 58.4	81.3 ± 66.7	

median (25th, 75th percentile)	108.7 (75.0, 161.8)	58.3 (37.7, 97.4)	52.9 (41.5, 79.4)	41.5 (25.7, 71.2)	74.8 (36.5, 159.2)	36.2 (22.8, 50.7)	49.7 (34.8, 103.8)	50.3 (33.4, 84.8)	50.7 (32.7, 79.3)	38.0 (25.8, 81.6)	31.9 (19.8, 49.6)	55.5 (24.1, 81.7)	54.2 (32.2, 127.1)	51.7 (29.3, 142.2)	
**3D SWE malignant (kPa)**															< .05^{F}
mean ± SD	142.4 ± 42.2	180.6 ± 50.4	158.9 ± 59.7	144.1 ± 56.6	178.9 ± 39.2	108.5 ± 8.4	111.9 ± 37.3	124.1 ± 36.4	89.1 ± 45.6	167.5 ± 25.2	152.5 ± 44.1	153.5 ± 62.1	144.0 ± 121.1	79.5 ± NA	
median (25th, 75th percentile)	151.1 (114.7, 164.5)	184.0 (146.8, 222.8)	172.2 (109.4, 206.2)	136.0 (95.3, 201.1)	173.4 (146.2, 214.3)	105.1 (102.3, NA)	101.0 (89.8, 124.1)	115.1 (96.7, 147.2)	85.5 (63.1, 104.2)	163.1 (146.8, 191.8)	150.1 (110.1, 196.9)	169.2 (110.7, 186.9)	144.0 (58.4, NA)	79.5 (79.5, 79.5)	

NA, not applicable.

^{F} F test (ANOVA); ^{C} Pearson's chi-squared test

$^{\#}$ Differences in the distribution of malignant and benign lesions among the study sites.

$^{\&}$ Differences in the distribution of BI-RADS 3, 4, and 5 lesions among the study sites.

Table S3. General distributions of benign and malignant lesions measured by 2D + 3D SWE and 2D SWE only.

Range of SWE (kPa)	2D SWE		2D + 3D SWE	
	Benign (%) n=581	Malignant (%) n=316	Benign (%) n=581	Malignant (%) n=316
**< 20**	62 (95.4)	3 (4.6)	31 (100)	0 (0)
**≥ 20 to < 30**	78 (95.1)	4 (4.9)	50 (96.1)	2 (3.8)
**≥ 30 to < 40**	117 (93.6)	8 (6.4)	70 (97.2)	2 (2.7)
**≥ 40 to < 50**	81 (89.0)	10 (11.0)	68 (97.1)	2 (2.9)
**≥ 50 to < 60**	68 (85.0)	12 (15.0)	74 (94.9)	4 (5.1)
**≥ 60 to < 70**	28 (70.0)	12 (30.0)	40 (90.9)	4 (9.1)
**≥ 70 to < 80**	26 (61.9)	16 (38.1)	45 (84.9)	8 (15.1)
**≥ 80 to < 90**	28 (52.8)	25 (47.2)	22 (61.1)	14 (38.9)
**≥ 90 to < 100**	16 (51.6)	15 (48.4)	15 (45.5)	18 (54.5)
**≥ 100 to < 110**	16 (53.3)	14 (46.7)	18 (62.1)	11 (37.9)
**≥ 110 to < 120**	5 (20.8)	19 (79.2)	21 (45.7)	25 (54.3)
**≥ 120 to < 130**	13 (54.2)	11 (45.8)	19 (55.9)	15 (44.1)
**≥ 130 to < 140**	3 (17.6)	14 (82.4)	6 (33.3)	12 (66.7)
**≥ 140 to < 150**	13 (54.2)	11 (45.8)	22 (61.1)	14 (38.9)
**≥ 150 to < 160**	7 (50.0)	7 (50.0)	7 (28.0)	18 (72.0)
**≥ 160 to < 170**	1 (10.0)	9 (90.0)	21 (55.3)	17 (44.7)
**≥ 170 to < 180**	2 (16.7)	10 (83.3)	18 (62.1)	11 (37.9)
**≥ 180 to < 190**	0 (0)	4 (100)	1 (9.1)	10 (90.9)
**≥ 190 to < 200**	2 (10.5)	17 (89.5)	9 (36.0)	16 (64.0)
**≥ 200 to < 210**	2 (28.6)	5 (71.4)	7 (41.2)	10 (58.9)
**≥ 210 to < 220**	3 (42.9)	4 (57.1)	1 (7.7)	12 (92.3)
**≥ 220 to < 230**	2 (25.0)	6 (75.0)	2 (22.2)	7 (77.8)
**≥ 230**	5 (5.7)	83 (94.3)	13 (13.0)	87 (87.0)

Figure S1. Representative image of 3D reconstruction in the axial (A), transverse (T) and coronal (C) planes in one case with a right breast lesion (fibroadenoma).

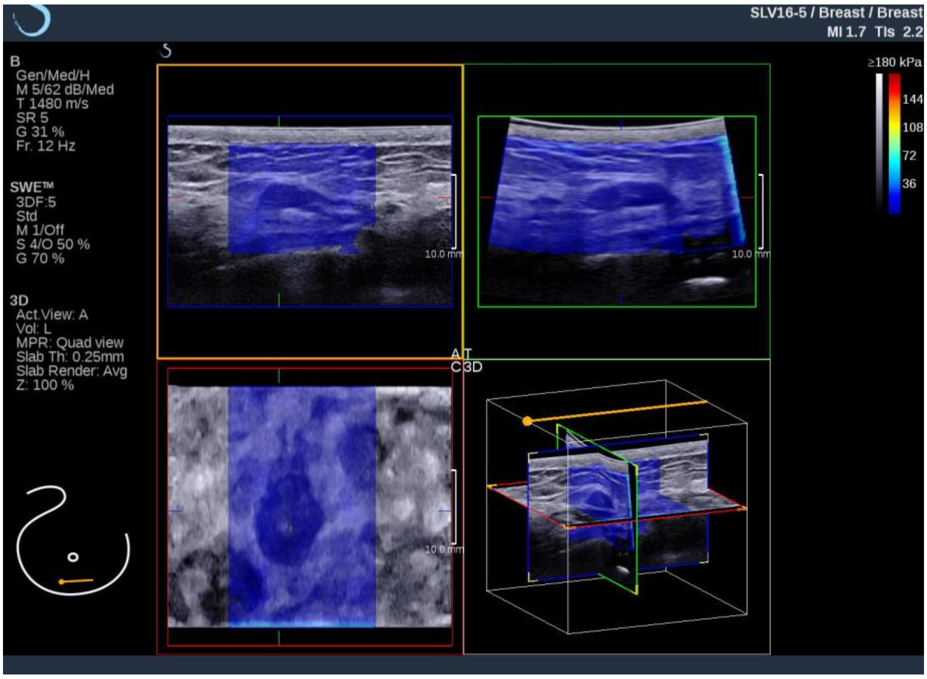

Figure S2. The stiffest part of the mass in the coronal image was automatically segmented into 4 x 4 slices based on the image processing software in the system.

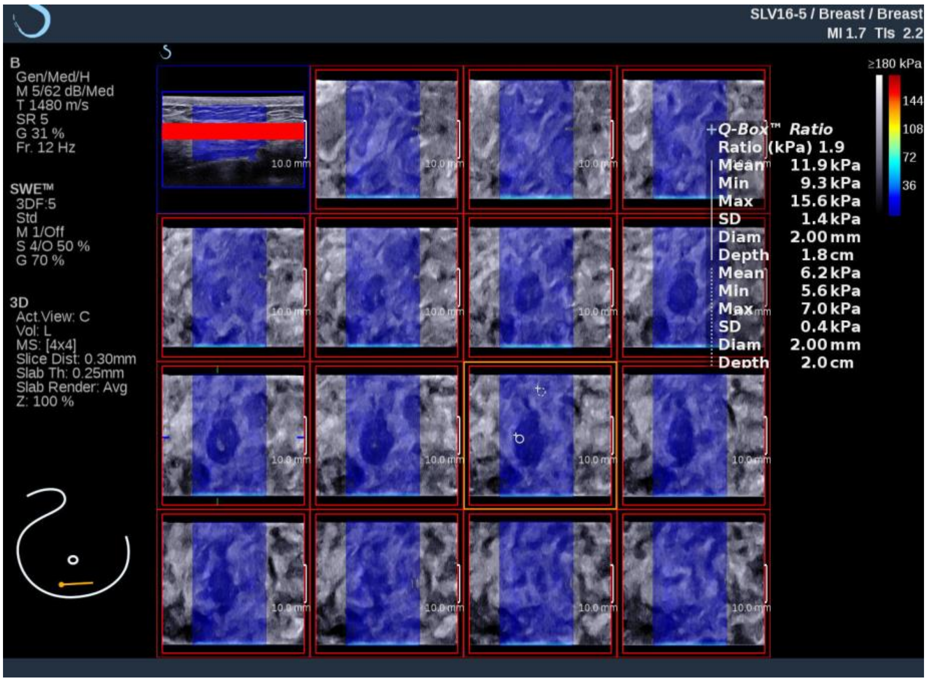

Supplement: Supplementary file 1 — (PDF 386 kb) [file 330_2023_10057_MOESM1_ESM.pdf]
